# Alpha1-antitrypsin ameliorates islet amyloid-induced glucose intolerance and β-cell dysfunction

**DOI:** 10.1016/j.molmet.2020.100984

**Published:** 2020-03-27

**Authors:** Júlia Rodríguez-Comas, Juan Moreno-Vedia, Mercè Obach, Carlos Castaño, Sara de Pablo, Gema Alcarraz-Vizán, Daniela Díaz-Catalán, Anna Mestre, Raquel Horrillo, Montserrat Costa, Anna Novials, Joan-Marc Servitja

**Affiliations:** 1 Diabetes and Obesity Research Laboratory, Institut d’Investigacions Biomèdiques August Pi i Sunyer (IDIBAPS), Barcelona, Spain; 2 Centro de Investigación Biomédica en Red de Diabetes y Enfermedades Metabólicas Asociadas (CIBERDEM), Spain; 3 Grifols Bioscience Research Group, Barcelona, Spain

**Keywords:** β-Cell, Macrophage, IAPP, Amyloid, Islet inflammation, Alpha1-antitrypsin, AAT, Alpha1-antitrypsin, T2D, Type 2 diabetes, hIAPP, Human islet amyloid polypeptide, hIAPP-Tg, Human islet amyloid polypeptide transgenic (mouse), GSIS, Glucose-stimulated insulin secretion, IL-1Ra, Interleukin-1 receptor antagonist, DIC, Dissociated islet cell, MФ, Macrophage

## Abstract

**Objective:**

Pancreatic β-cell failure is central to the development and progression of type 2 diabetes (T2D). The aggregation of human islet amyloid polypeptide (hIAPP) has been associated with pancreatic islet inflammation and dysfunction in T2D. Alpha1-antitrypsin (AAT) is a circulating protease inhibitor with anti-inflammatory properties. Here, we sought to investigate the potential therapeutic effect of AAT treatment in a mouse model characterized by hIAPP overexpression in pancreatic β-cells.

**Methods:**

Mice overexpressing hIAPP (hIAPP-Tg) in pancreatic β-cells were used as a model of amyloid-induced β-cell dysfunction. Glucose homeostasis was evaluated by glucose tolerance tests and insulin secretion assays. Apoptosis and amyloid formation was assessed in hIAPP-Tg mouse islets cultured at high glucose levels. Dissociated islet cells were cocultured with macrophages obtained from the peritoneal cavity.

**Results:**

Nontreated hIAPP-Tg mice were glucose intolerant and exhibited impaired insulin secretion. Interestingly, AAT treatment improved glucose tolerance and restored the insulin secretory response to glucose in hIAPP-Tg mice. Moreover, AAT administration normalized the expression of the essential β-cell genes *MafA* and *Pdx1*, which were downregulated in pancreatic islets from hIAPP-Tg mice. AAT prevented the formation of amyloid deposits and apoptosis in hIAPP-Tg islets cultured at high glucose concentrations. Since islet macrophages mediate hIAPP-induced β-cell dysfunction, we investigated the effect of AAT in cocultures of macrophages and islet cells. AAT prevented hIAPP-induced β-cell apoptosis in these cocultures without reducing the hIAPP-induced secretion of IL-1β by macrophages. Remarkably, AAT protected β-cells against the cytotoxic effects of conditioned medium from hIAPP-treated macrophages. Similarly, AAT also abrogated the cytotoxic effects of exogenous proinflammatory cytokines on pancreatic β-cells.

**Conclusions:**

These results demonstrate that treatment with AAT improves glucose homeostasis in mice overexpressing hIAPP and protects pancreatic β-cells from the cytotoxic actions of hIAPP mediated by macrophages. These results support the use of AAT-based therapies to recover pancreatic β-cell function for the treatment of T2D.

## Highlights

• Alpha1-antitrypsin (AAT) ameliorates glucose intolerance in hIAPP transgenic mice.
• AAT improves insulin secretion in hIAPP transgenic mice.
• AAT prevents apoptosis and amyloid deposition in cultured hIAPP transgenic islets.
• AAT protects β-cells from hIAPP-induced cytotoxicity mediated by macrophages.
• AAT abrogates the cytotoxic effects of proinflammatory cytokines on β-cells.

## Graphical abstract

**Figure:**
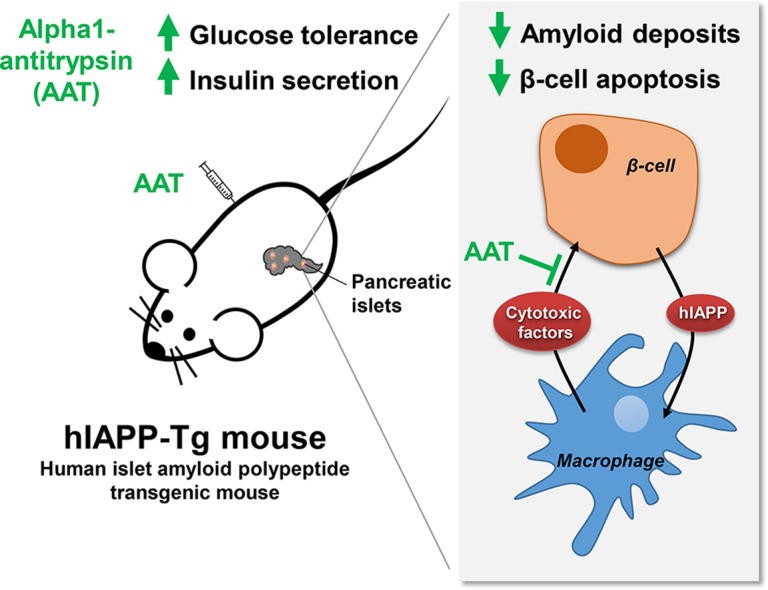

## 1 Introduction

Patients with type 2 diabetes mellitus (T2D) are unable to secrete sufficient insulin to compensate for increased peripheral insulin resistance. Pancreatic β-cell failure is central to the development and progression of T2D. Pancreatic islets in T2D are characterized by a deficit in β-cell mass, impaired β-cell function, and the presence of extracellulr amyloid deposits [[1], [2], [3]]. These amyloid deposits are mainly composed of islet amyloid polypeptide (IAPP), a hormone that is produced and secreted as a monomeric form with insulin by β-cells [4]. The human form of IAPP (hIAPP) has a propensity to form aggregates and fibrils that are cytotoxic to β-cells [5,6]. In contrast to hIAPP, rodent IAPP does not aggregate and, therefore, is nonamyloidogenic.

One of the mechanisms by which hIAPP aggregation is associated with β-cell dysfunction and death is through the induction of islet inflammation [[7], [8], [9], [10]]. hIAPP accumulation within the pancreatic islets activates macrophages via the inflammasome and induces the secretion of proinflammatory cytokines (mainly IL-1β), which, in turn, contribute to β-cell toxicity [11].

Anti-inflammatory agents represent a promising approach to treating the low-grade inflammation commonly found in T2D subjects. It has been shown that drugs blocking IL-1β such as IL-1 receptor antagonists (IL-1Ra) or anti-IL-1β antibodies can preserve β-cell function in T2D patients [12,13] and reduce islet inflammation in the type 2 diabetic GK rat [14]. In this context, blockade of IL-1β has been shown to ameliorate amyloid-induced glucose intolerance and islet inflammation [9] and to increase β-cell survival after islet transplantation [15].

Alpha1-antitrypsin (AAT) is an abundant circulating serine protease inhibitor that has been proven to be a safe drug and has effectively been used for long-term treatment of pulmonary emphysema in patients with hereditary deficiency of AAT for decades [16]. Besides the inhibition of proteases such as neutrophil elastase, AAT exerts anti-inflammatory and antiapoptotic actions. Several studies have demonstrated protective effects of AAT on pancreatic islets in different experimental models of type 1 diabetes (T1D) and islet transplantation. Thus, AAT has been shown to prolong islet allograft survival in mice [[17], [18], [19]], prevent the onset of T1D in nonobese diabetic mice [20], and protect β-cells from apoptosis *in vivo* [21]. However, whether AAT treatment may also exert a therapeutic action by rescuing the β-cell function in T2D models remains unknown. To address this, we explored the potential protective effect of AAT in transgenic mice with overexpression of hIAPP in pancreatic β-cells, which results in islet dysfunction and glucose intolerance.

## 2 Materials and methods

*Experimental Animal Model.* Heterozygous male transgenic mice with β-cell-specific expression of hIAPP (FVB/N-Tg (Ins2-IAPP)RHFSoel/J) were purchased from The Jackson Laboratory. Nontransgenic wild-type (WT) littermates were used as controls. Mice aged 6 weeks were intraperitoneally injected three times per week with human AAT (Prolastin®-C, Grifols) until 16 weeks of age. The initial dose of AAT was 2 mg/mouse for the first 2 weeks, and then, the dose was gradually increased by 0.5 mg/mouse with each injection until reaching a maximum dose of 6 mg/mouse. Protocols were approved by the Animal Ethics Committee of the Universitat de Barcelona, and the Principles of Laboratory Animal Care were followed.

*Intraperitoneal Glucose Tolerance Tests (IPGTTs) and Insulin Tolerance Tests (ITTs)*. IPGTTs were performed by intraperitoneal injection of d-glucose (2 g/kg body weight) in hIAPP transgenic (hIAPP-Tg) and WT mice unfed for 16 h. Glucose levels in tail vein blood samples were measured with a blood glucometer (Nova Pro) at different time points after glucose injection. Blood samples for insulin determination were collected through the tail vein just before the injection of glucose and 15 min after injection. Plasma insulin levels were measured using an ELISA kit (Crystal Chem). For ITTs, 0.7 U/Kg human insulin (Novo Nordisk) was intraperitoneally injected and glycemia was measured at different time points.

*Islet Isolation and Culture*. Mouse pancreatic islets were isolated from C57BL/6J male mice and from hIAPP-Tg mice and nontransgenic littermates by collagenase digestion and Histopaque gradient (Sigma–Aldrich) as described elsewhere [22]. Islets were allowed to recover for 24 h at 37 °C and 5% CO2 in RPMI 1640 medium (11.1 mM glucose) supplemented with 10% fetal bovine serum (FBS) (v/v), 2 mM glutamine, 100 U/mL penicillin, and 100 μg/mL streptomycin. AAT (0.5 mg/mL) was added after the overnight recovery and was maintained throughout the experiment. C57BL/6J islets were challenged with a combination of cytokines (IL-1β (1 U/mL), IFN-γ (20 U/mL), and TNFα (20 U/mL); R&D systems) for 24 h. hIAPP-Tg and WT islets were cultured for 2–7 days in RPMI at 11.1 mM or 16.7 mM glucose. Dissociated islet cells (DICs) were obtained from isolated C57BL/6J islets and maintained as described elsewhere [23]. The IL-1 receptor antagonist anakinra (PeproTech) was dissolved in water and used at 4 μg/mL.

*Peritoneal Macrophages*. 10 mL of sterile PBS was intraperitoneally injected into C57BL/6J mice and the peritoneal cavity content was aspirated. The aspirates were centrifuged, washed, and seeded into 96-well plate (100.000 cells/well) in RPMI 1640 medium supplemented with 10% FBS (v/v), 2 mM glutamine, 100 U/mL penicillin, and 100 μg/mL streptomycin. After an overnight recovery, peritoneal macrophages (pMФ) were primed with LPS (5 ng/mL) (Sigma–Aldrich) for 3 h. hIAPP (Bachem) was dissolved in water and rapidly added to the medium at a final concentration of 10 μM. The purity of peritoneal macrophage cultures was determined by CD68 immunostaining.

*Gene Expression Analysis*. Total RNA from pancreatic islets was extracted using the miRNeasy Kit (Qiagen, Hilden, Germany) and reverse-transcribed using SuperScript (Invitrogen). Quantitative PCR was performed using SYBR Green (Invitrogen) in a 7900HT Fast Real-Time PCR system (Applied Biosystems). Expression levels were normalized to the expression of an endogenous house-keeping gene (*Hprt1*). See Supplementary Table S1 for primer sequences.

*Immunohistochemistry*. Islets and DICs were fixed for 15 min with 10% formalin and then were permeabilized with 0.2% Triton X-100 and blocked with 1% BSA. For *in toto* immunostaining, islets were permeabilized with 0.5% Triton X-100 (Sigma–Aldrich) and blocked with 10% FBS in PBS. Samples were incubated with polyclonal guinea pig anti-insulin (1:500; Dako) and rabbit anti–cleaved caspase-3 (1:400; Cell Signaling Technology) antibodies, followed by secondary incubation with Alexa Fluor 555 conjugated goat anti–guinea pig IgG (1:250; Life Technologies) and Donkey anti-Rabbit Alexa Fluor 488 (1:250; Jackson IR). For double insulin and amyloid (thioflavin S) staining, sections of isolated pancreatic islets embedded in 2% agarose were stained for insulin as described above, followed by incubation in 0.5% thioflavin S (Sigma–Aldrich) solution for 2 min and rinsed twice with 70% ethanol. TUNEL staining was performed in agarose-embedded islets and in DICs using the DeadEnd Fluorometric TUNEL system (Promega). Hoechst 33342 (Sigma–Aldrich) was used to stain nuclei. Images were taken with an epifluorescence microscope (DMR HC, Leica Microsystems) and, for *in toto* immunostaining, with a Leica TCS SPE confocal microscope. Fluorescence images were obtained using Leica LAS Image Analysis software. ImageJ version 1.49 software (National Institutes of Health) was used to determine the islet area, insulin-positive area, and thioflavin staining.

*Statistical Analysis.* Data are expressed as mean ± SEM. The D'Agostino–Pearson omnibus normality test was carried out to test whether the variables followed a normal distribution. Statistical significance was determined by the unpaired Student's *t-*test for parametric quantitative variables and the Mann–Whitney *U* test for quantitative nonparametric variables when comparing two groups. One-way or two-way ANOVA with post hoc Tukey's test was used when comparing more than two groups. A value of *P* < 0.05 was considered statistically significant.

## 3 Results

**Figure 1:**
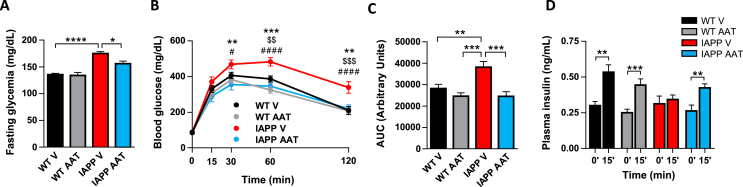
*In vivo* treatment with alpha1-antitrypsin (AAT) normalizes glucose homeostasis and improves insulin secretion in hIAPP-Tg mice. WT and hIAPP-Tg mice (IAPP) were treated intraperitoneally with vehicle (V) or AAT (2–6 mg/mouse) from 6 to 16 weeks of age. At the end of the treatment, different parameters related to glucose homeostasis were determined. (A) Glycemia after 5 h of fasting. (B–C) Intraperitoneal glucose tolerance test (IPGTT) in mice after 10 weeks of AAT or V intraperitoneal injections. Glycemia values and respective areas under the curve (AUC) are shown. ∗*P* < 0.05; ∗∗*P* < 0.01; ∗∗∗*P* < 0.001; ∗∗∗∗*P* < 0.0001 IAPP/V versus IAPP/AAT. $$ *P* < 0.01; $$$ *P* < 0.001 IAPP/V versus WT/V. #*P* < 0.05; ####*P* < 0.001 IAPP/V versus WT/AAT. (D) Plasma insulin levels at 0 min and 15 min after glucose injection. Results are expressed as the mean ± SEM from two independent experiments (n = 9–16 mice/group). ∗*P* < 0.05; ∗∗*P* < 0.01; ∗∗∗*P* < 0.001; ∗∗∗∗*P* < 0.0001.

***AAT Treatment Restores Glucose Homeostasis in hIAPP Transgenic Mice***. To explore a potential protective effect of AAT in hIAPP transgenic (hIAPP-Tg) mice *in vivo*, AAT or a vehicle solution was administered intraperitoneally three times per week (2–6 mg/mouse) for 10 weeks in hIAPP-Tg mice and nontransgenic littermates (the latter incapable of forming islet amyloid). AAT treatment did not alter body weight and food intake (data not shown). Pharmacokinetic analysis revealed increased levels of human AAT (hAAT) and antielastase activity in the serum of AAT-treated mice along the treatment period (Supplementary Figure S1). At the end of the experiment (16 weeks of age), glucose homeostasis was evaluated in the four experimental groups. Vehicle-treated hIAPP-Tg mice exhibited increased glycemia after 5-hour fasting that was normalized in AAT-treated hIAPP-Tg mice (Figure 1A). As expected, hIAPP-Tg mice were clearly glucose intolerant at 16 weeks of age. Strikingly, AAT treatment completely restored glucose tolerance (Figure 1B,C). To test the secretory capacity of β-cells *in vivo*, insulin levels were determined in plasma samples of mice 15 min after an intraperitoneal injection of glucose. Glucose-induced insulin secretion was severely blunted in hIAPP-Tg mice. Remarkably, insulin secretion was completely restored in AAT-treated hIAPP-Tg mice (Figure 1D). Taken together, these results revealed that AAT treatment restores glucose homeostasis in hIAPP-Tg mice.

In order to test whether the AAT-induced improvement of glucose tolerance was due to an effect on insulin sensitivity, we performed ITTs. As expected from our own previous studies and from other groups [8,9,24] hIAPP-Tg mice did not exhibit insulin resistance and, importantly, AAT administration had no impact on the ITT in both WT and hIAPP-Tg mice (Supplementary Figure S2). This, together with the improvement in insulin secretion, supports that AAT improves glucose tolerance mainly by ameliorating the detrimental effects of hIAPP on pancreatic islets.

**Figure 2:**
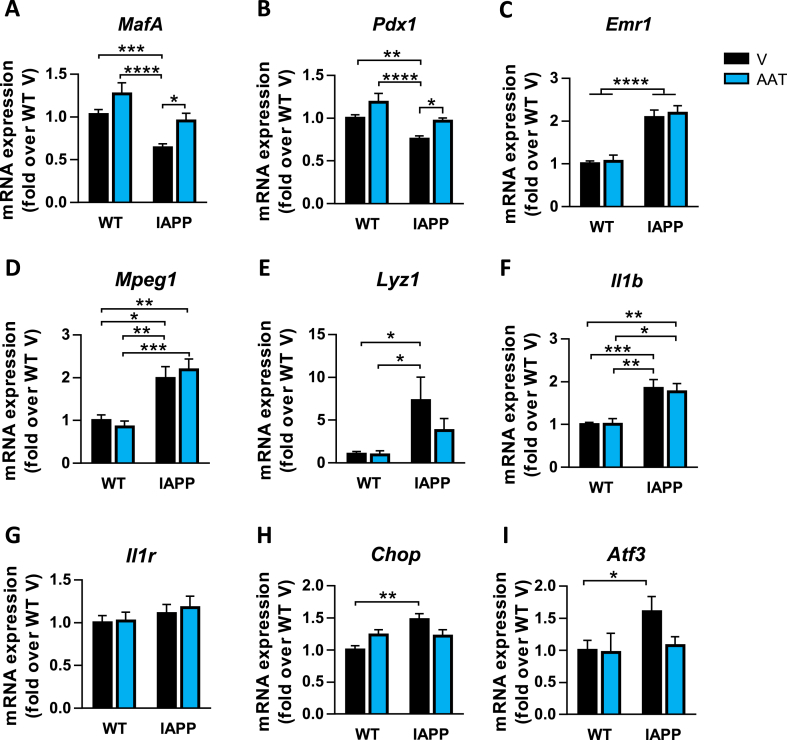
Effects of AAT treatment on gene expression in pancreatic islets from hIAPP-Tg mice. (A–I) mRNA expression levels of (A) *MafA*, (B) *Pdx1*, (C) *Emr1*, (D) *Mpeg1*, (E) *Lyz1*, (F) *Il1b*, (G) *Il1r*, (H) *Chop*, and (I) *Atf3* in pancreatic islets isolated from WT and hIAPP-Tg (IAPP) mice treated with vehicle (V) or AAT. Gene expression data were normalized against Hprt1 and are shown relative to WT-V levels, which were set arbitrarily to 1. Results are expressed as the mean ± SEM from two independent experiments (n = 9–16 mice/group). ∗*P* < 0.05; ∗∗*P* < 0.01; ∗∗∗*P* < 0.001; ∗∗∗∗*P* < 0.0001.

Despite the systematic glucose intolerance that we observe in hIAPP transgenic mice, we did not observe significant morphometric abnormalities or increased β-cell apoptosis in the pancreases of these mice. In order to gain more insights into the effects of AAT, we next focused on the gene profile of pancreatic islets from WT and hIAPP-Tg mice treated or not with AAT. We first evaluated genes encoding key transcription factors involved in the β-cell function and insulin expression such as *MafA* and *Pdx1*. The expression of these genes was significantly decreased in islets from hIAPP-Tg mice and, remarkably, AAT treatment prevented this reduction (Figure 2A,B). Consistent with the fact that hIAPP overexpression results in islet inflammation and macrophage infiltration, the expression of the macrophage markers *Emr1* (F4/80), *Mpeg1*, and *Lyz1* was upregulated in hIAPP-Tg mice islets (Figure 2C–E). In line with this, the expression of the gene encoding the proinflammatory cytokine Il-1β (*Il1b*) followed the same pattern rather than the macrophage marker genes (Figure 2F). However, the expression of these genes was not affected by AAT treatment, suggesting that the number of macrophages was maintained. The expression levels of the IL-1 receptor gene (*Il1r*) were not altered in transgenic mice and were not modified by AAT treatment (Figure 2G). In contrast, AAT partially ameliorated the upregulation of the stress genes *Chop* and *Atf3*, which were slightly upregulated in hIAPP-Tg mice (Figure 2H,I). Collectively, these results show that AAT treatment has a significant effect on hIAPP-Tg mouse islet by preventing the downregulation of key genes for β-cell function, without a significant effect on macrophage and inflammation markers.

**Figure 3:**
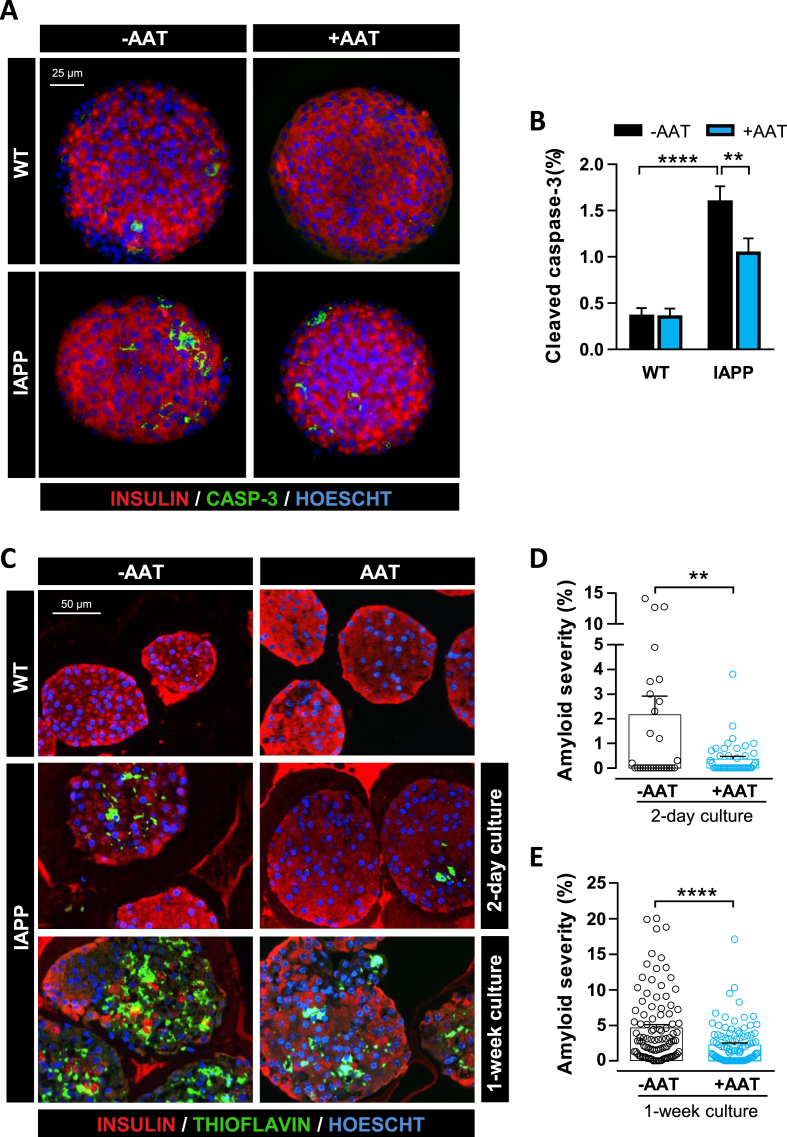
AAT treatment reduces apoptosis and amyloid accumulation in hIAPP-Tg islets cultured at high glucose. (A) *In toto* immunostaining for insulin (red), cleaved caspase-3 (green), and Hoechst (blue) of WT and hIAPP-Tg islets (IAPP) cultured 2 days at 16.7 mM glucose, in the absence (-AAT) or the presence (+AAT) of AAT (0.5 mg/mL). Scale bar, 25 μm. (B) Quantification of cleaved caspase-3-positive cells relative to the total number of islet cells in WT and hIAPP-Tg islets (IAPP) treated as in A. (C) Representative images of amyloid formation in WT and hIAPP-Tg islets cultured at 16.7 mM glucose for 2 days or 1 week, a setting that prompts amyloid deposition in hIAPP-Tg islets, in the absence (-AAT) or the presence (+AAT) of AAT. No amyloid formation was observed in WT islets, used here as negative controls. Insulin (red), Hoechst (blue), and amyloid (green) were determined by immunostaining and thioflavin S staining. Scale bar, 50 μm. (D, E) Quantification of amyloid severity (percentage of amyloid area with respect to total islet area) in islets treated as in C. Three different preparations of islets, from three mice each, were used. Individual results for each islet are displayed. Results are expressed as the mean ± SEM of 98 (-AAT) and 96 (+AAT) islets cultured for 2 days (D), and 29 (-AAT) and 44 (+AAT) islets cultured for 7 days (E). ∗*P* < 0.05; ∗∗*P* < 0.01; ∗∗∗*P* < 0.001; ∗∗∗∗*P* < 0.0001. (For interpretation of the references to color in this figure legend, the reader is referred to the Web version of this article.)

***AAT Treatment Reduces Amyloid Deposition and Apoptosis in hIAPP Islets Ex Vivo***. To assess the protective effect of AAT against β-cell dysfunction induced by endogenously produced hIAPP, islets from WT and hIAPP-Tg mice were isolated and cultured at high glucose (16.7 mM) levels. This condition accelerates hIAPP aggregation and the formation of extracellular amyloid deposits, providing an experimental model to explore these events. We first analyzed apoptosis in pancreatic islets cultured for 2 days by immunostaining of cleaved caspase-3. We observed an increased percentage of apoptosis in hIAPP-Tg islets (1.6 ± 0.2% of cleaved caspase-3-positive cells with respect to the total number of cells) compared to WT islets (0.4 ± 0.1%) and, remarkably, AAT reduced apoptosis in transgenic islet cells by 34.6% (Figure 3A,B). Consistent with the apoptotic rate observed within hIAPP-Tg islets cultured at 16.7 mM glucose, we also observed accumulation of extracellular amyloid deposits, detected by thioflavin S staining. As shown in Figure 3C, amyloid deposits were progressively formed throughout hIAPP islets, engaging 2.2 ± 0.8% and 4.6 ± 0.5% of the islet area after 2 and 7 days of culture at high glucose levels, respectively. Interestingly, when islets were cultured in the presence of AAT, amyloid severity was reduced 6-fold after 2 days in culture (Figure 3C,D). Even after 7 days of culture, when amyloid severity was very high, AAT reduced these levels more than 2-fold. Of note, the frequency of islets exhibiting high amyloid severity (>5%) after 7 days of culture was reduced from 33.7% to 11.6% in the presence of AAT (Figure 3C,E). Altogether, these results show that AAT blocks hIAPP-induced cell apoptosis and amyloid formation in pancreatic islets, which may be central to the observed beneficial effects of AAT treatment on glucose homeostasis and insulin secretion.

**Figure 4:**
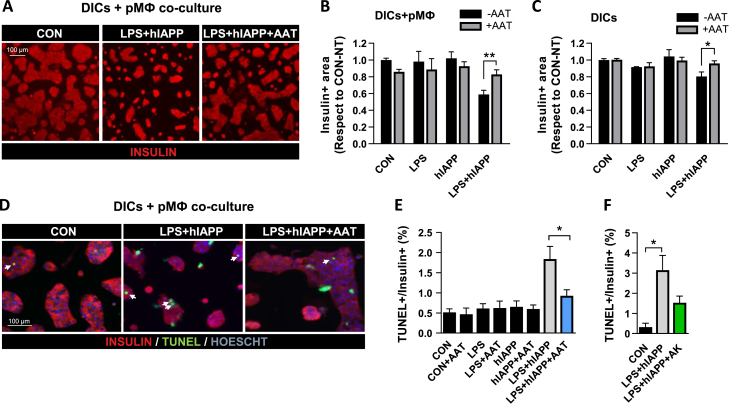
AAT attenuates hIAPP-triggered β-cell growth reduction and β-cell apoptosis in DICs cocultured with macrophages. (A) Representative images of the insulin-positive area of DICs cocultured with peritoneal macrophages (pMФ) for 72 h and treated (LPS + hIAPP) or not (CON) for the last 24 h with LPS (5 ng/mL) and exogenous hIAPP (10 μM) in the presence (+AAT) or the absence (-AAT) of AAT (0.5 mg/mL). Scale bar, 100 μm. (B) Quantification of the insulin-positive area of DICs cocultured with peritoneal macrophages (pMФ) for 72 h and treated or not (CON) for the last 24 h with LPS, exogenous hIAPP, or the combination of LPS and exogenous hIAPP (LPS + hIAPP) in the presence or the absence of AAT. Quantifications are shown relative to the nontreated control samples (CON, -AAT), which were arbitrarily set to 1. (C) Quantification of the insulin-positive area of DICs (without peritoneal macrophages) cultured for 72 h and treated as in B. (D) Representative images of TUNEL (green), Hoescht (blue), and insulin (red) staining of DICs cocultured with peritoneal macrophages. Wells were treated as in A. The arrows point to apoptotic β-cells identified by TUNEL staining. Scale bar, 100 μm. (E) Quantification of β-cell apoptosis in cocultures of DICs and peritoneal macrophages and treated as in B. β-cell apoptosis was calculated as the percentage of TUNEL- and insulin-positive with respect to the total number of insulin-positive cells. (F) Quantification of β-cell apoptosis in cocultures of DICs and peritoneal macrophages and treated with LPS and hIAPP in the presence (+AK) or the absence of the IL-1R antagonist anakinra used at 4 μg/mL. Results are expressed as the mean ± SEM from at least three independent experiments, each one including at least two different replicates per condition. ∗*P* < 0.05; ∗∗*P* < 0.01; ∗∗∗*P* < 0.001; ∗∗∗∗*P* < 0.001. (For interpretation of the references to color in this figure legend, the reader is referred to the Web version of this article.)

***AAT Protects Pancreatic β-Cells against hIAPP-Induced Cytotoxicity Mediated by Macrophages***. hIAPP acts as a potent stimulus for macrophages to secrete proinflammatory cytokines such as IL-1β [9,11], which can, in turn, compromise islet function and lead to β-cell death [25,26] and dedifferentiation [27]. Indeed, resident macrophages have been shown to mediate hIAPP-induced β-cell dysfunction through IL-1β secretion [8]. To explore the protective role of AAT against macrophage-mediated β-cell dysfunction induced by hIAPP, peritoneal macrophages were cocultured with DICs from C57BL/6J mice with an islet cell:macrophage ratio of 50:1. Processing of IL-1β initiated by hIAPP first requires priming and activation of the NLRP3 inflammasome by LPS [11]. For this reason, cocultures were treated with a very low dose of LPS (5 ng/mL) before adding hIAPP. After 72 h of LPS and hIAPP addition, we analyzed the β-cell area and apoptosis. hIAPP addition in the presence of LPS-primed macrophages resulted in a reduction of over 40% in the β-cell area with respect to control cocultures. Remarkably, this decrease in β-cell growth was blocked in the presence of AAT (Figure 4A,B). Of note, the effect of LPS and hIAPP was lower in the absence of peritoneal macrophages (Figure 4C), illustrating the role of macrophages in amplifying the detrimental effects of hIAPP on β-cells. It is also important to note that LPS or hIAPP alone had no effects on β-cell growth both in cocultures and in DICs alone (Figure 4B,C). In line with the impairment of β-cell growth, TUNEL-positive β-cells increased more than 3-fold in cocultures treated with LPS and hIAPP compared to nontreated cocultures, and AAT treatment almost completely blocked hIAPP-induced apoptosis (Figure 4D,E). Again, LPS or hIAPP alone did not have an impact on β-cell apoptosis, even in the presence of peritoneal macrophages (Figure 4E). Treatment with an IL-1R antagonist (anakinra) also reduced TUNEL-positive β-cells, corroborating that IL-1β is the main player in hIAPP-induced β-cell failure (Figure 4F). Taken together, these results uncovered a protective role of AAT on β-cells against hIAPP-activated macrophages.

**Figure 5:**
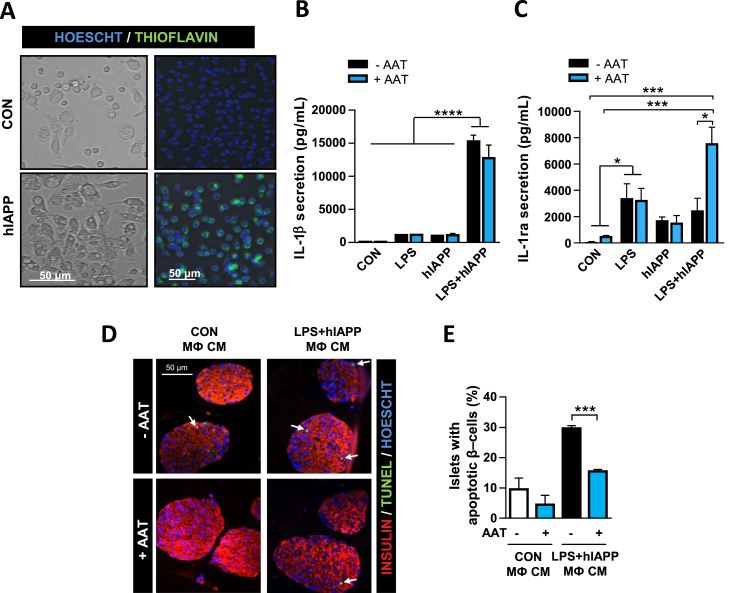
AAT protects pancreatic β-cells against conditioned medium from hIAPP-treated macrophages. (A) Cultured peritoneal macrophages nontreated (CON) or treated with exogenous hIAPP (10 μM). Macrophages under the light microscope are shown on the left. The right panels show fixed macrophages stained with thioflavin S to detect amyloid fibrils. Scale bars, 50 μm. Of interest, amyloid staining is found inside the macrophages. (B) IL-1β secretion from peritoneal macrophages previously primed or not for 3 h with LPS (5 ng/mL), treated or not with hIAPP (10 μM) and in the presence (+AAT) or the absence (-AAT) of AAT (0.5 mg/mL). Nonstimulated peritoneal macrophages were used as controls (CON). (C) IL-1 receptor antagonist (IL-1Ra) secretion from macrophages treated as in B. (D) Conditioned medium (CM) from cultured peritoneal macrophages (pMФ) previously treated as in B (without AAT) was added to islets from C57BL/6J mice. AAT (0.5 mg/mL) was added or not to cultured islets 1 h before the addition of the CM. Images are representative of islets stained for insulin (red), TUNEL (green), and Hoechst (blue). The arrows point to apoptotic β-cells identified by TUNEL and insulin staining. Scale bar, 50 μm. (E) Quantification of islets, treated as in D, presenting β-cell apoptosis (at least one TUNEL- and insulin-positive cell). Results are expressed as the mean ± SEM from three independent experiments. ∗*P* < 0.05; ∗∗∗*P* < 0.001; ∗∗∗∗*P* < 0.0001. (For interpretation of the references to color in this figure legend, the reader is referred to the Web version of this article.)

***AAT Protects Pancreatic β-Cells from the Cytotoxic Effects of hIAPP-Treated Macrophages***. We next sought to determine whether AAT may protect β-cells from cytotoxic factors released by hIAPP-treated macrophages. This protection could be achieved by attenuating macrophage activation and/or by protecting pancreatic β-cells against macrophage-derived factors. First, we explored whether AAT was able to reduce hIAPP-induced IL-1β secretion by peritoneal macrophages. Exogenously added hIAPP was incorporated by macrophages and resulted in a dramatic change of their morphology (Figure 5A). Both LPS and hIAPP triggered an increase of IL-1β secretion, which was synergistically potentiated when hIAPP was added to LPS-primed macrophages. This is in line with the previous results showing that LPS and hIAPP together induced a potent reduction of the β-cell area associated with an increase in apoptosis in cocultures of DICs and macrophages, whereas LPS or hIAPP alone did not have any effect. Remarkably, IL-1β secretion induced by hIAPP in LPS-primed macrophages was unaffected by AAT (Figure 5B). Gene expression of *Il1b* in hIAPP-treated macrophages was also unaffected by AAT treatment (Supplementary Figure S3). Similarly, AAT did not modify hIAPP-triggered induction of genes encoding other proinflammatory factors such as TNF-α (*Tnfa*), Ccl2 (*Ccl2*), and Nlrp3 (*Nlrp3*) or the anti-inflammatory molecules IL1-R antagonist (*Ilrn*) and IL-10 (*Il10*) (Supplementary Figure S3). Nevertheless, AAT administration led to an increase in IL-1Ra levels in the medium of LPS-primed macrophages stimulated with hIAPP (Figure 5C), which may help to resolve islet inflammation.

Then, we addressed whether AAT was able to protect β-cells against macrophage-secreted factors. For this purpose, conditioned medium from nontreated macrophages or LPS-primed macrophages treated with hIAPP was added to isolated pancreatic islets for 24 h. In the presence of this conditioned medium, the frequency of islets showing at least one TUNEL-positive β-cell increased from 9.6 ± 3.7% in islets treated with control medium to 29.7 ± 0.9% in islets treated with medium from hIAPP-treated macrophages (Figure 5D). Remarkably, the number of islets with apoptotic β-cells in this conditioned medium was reduced to 15.5 ± 0.7% when AAT was added to the islets (Figure 5E), revealing a protective role of AAT on islet cells against macrophage-secreted factors.

**Figure 6:**
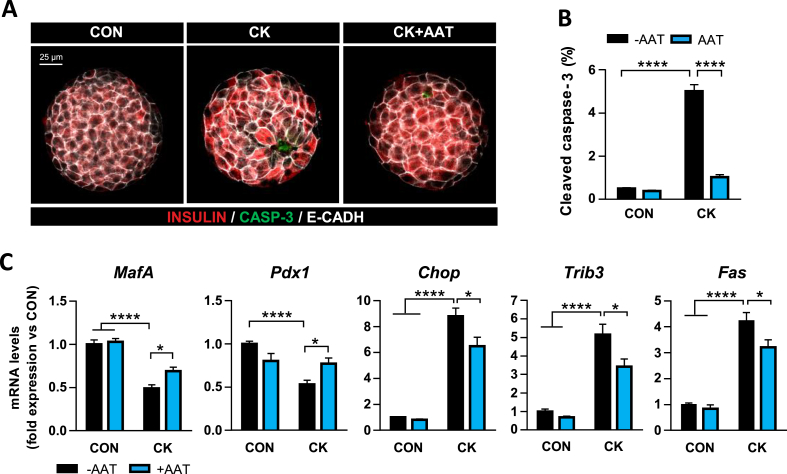
AAT protects pancreatic islet cells against proinflammatory cytokines. (A) *In toto* immunostaining for insulin (red), cleaved caspase-3 (green), and E-cadherin (white) of isolated pancreatic islets after 24 h of treatment with a combination of proinflammatory cytokines (CK: IL-1β (1 U/mL), IFNγ (20 U/mL), and TNFα (20 U/mL)), in the presence or the absence of AAT (0.5 mg/mL). AAT was added 1 h prior to the addition of cytokines. Scale bar, 25 μm. (B) Quantification of cleaved caspase-3-positive cells with respect to the total number of islet cells. Results are expressed as the mean ± SEM from 10 to 25 islets in three independent experiments. (C) mRNA expression levels of *MafA*, *Pdx1*, *Chop*, *Trib3*, and *Fas* in mouse islets after 24 h of treatment with cytokines (CK) as in A. Gene expression data were normalized against *Hprt1* and are shown relative to nontreated control islets (CON), which were set arbitrarily to 1 (n = 7–11). ∗*P* < 0.05; ∗∗∗*P* < 0.001; ∗∗∗∗*P* < 0.0001. (For interpretation of the references to color in this figure legend, the reader is referred to the Web version of this article.)

***AAT Prevents Cytokine-Induced Apoptosis and Stress in Pancreatic Islets***. Our observation that AAT protects against extracellular factors secreted by hIAPP-treated macrophages prompted us to assess whether AAT was also effective in protecting β-cells from exogenously administered proinflammatory cytokines. Isolated pancreatic islets from wild-type C57BL/6J mice were exposed to a combination of proinflammatory cytokines including IL-1β (1 U/mL), IFN-γ (20 U/mL), and TNFα (20 U/mL) for 24 h. The concentrations of these cytokines were lower than those used in other settings that try to mimic the islet environment in the context of T1D [28] as we sought to mimic a mild inflammatory milieu. Immunostaining of cleaved caspase-3 revealed that cytokines induced apoptosis in 5.0 ± 0.3% of islet cells. Importantly, AAT prevented about 80% of cytokine-induced apoptosis in pancreatic islets (Figure 6A,B). We also explored the impact of AAT on the changes in gene expression triggered by cytokines. As observed in hIAPP-Tg islets, AAT treatment prevented the loss of β-cell identity markers *MafA* and *Pdx1* in islets exposed to cytokines (Figure 6C). The effect of cytokines on these β-cell genes was accompanied by the induction of endoplasmic reticulum stress genes such as *Chop* and *Trib3*, as well as the apoptotic gene *Fas*. AAT significantly reduced the induction of these stress and apoptotic genes by cytokines (Figure 6C). These findings reveal that AAT protects pancreatic β-cells against the cytotoxic actions of proinflammatory cytokines.

## 4 Discussion

β-cell failure is central to the development and progression of T2D, and therapeutic strategies aimed at rescuing pancreatic β-cell function are emerging in T2D treatment. Compelling evidence suggests that aggregation of hIAPP contributes to islet inflammation and dysfunction in T2D [1,3,9,11,24,29].

In the present study, we describe a novel potential therapeutic action of AAT for the treatment of T2D. AAT is a circulant serine protease inhibitor that exerts anti-inflammatory and immunomodulatory properties [30]. Most of the studies on AAT involving pancreatic β-cells have addressed T1D and islet transplantation, focusing on potential beneficial effects of AAT in modulating the immune response [18,20]. Here, we show that systemic administration of AAT restores glucose homeostasis in transgenic mice with overexpression of hIAPP in β-cells, which results in glucose intolerance and impaired insulin secretion. Furthermore, AAT treatment also normalizes the expression of the genes encoding Pdx1 and MafA, two transcription factors essential for β-cell function and whose expression is regularly altered in pancreatic islets under stress conditions. Indeed, loss of such β-cell identity markers has been recently proposed as a mechanism underlying β-cell failure in T2D [27].

The fact that hIAPP-Tg mice exhibit normal insulin sensitivity [8,24] suggests that the beneficial effects of AAT are mediated by a direct action on islet cells. In this context, our results show that AAT reduces islet cell apoptosis and attenuates the formation of amyloid deposits in hIAPP-Tg islets cultured *ex vivo* at high glucose. Of note, apoptosis was detected in islets cultured 2 days at high glucose, but not after 7 days of culture, when high amounts of amyloid deposits were detected. This is consistent with *in vivo* time-course studies illustrating that hIAPP-Tg animals show the highest frequency of β-cell apoptosis at 10 weeks of age, when almost no amyloid is formed, and the frequency decreases at 40 weeks, when islet amyloid reaches the highest levels [31]. Moreover, our results are in line with the notion that toxic hIAPP oligomers, rather than hIAPP fibrils or amyloid deposits, initiate β-cell apoptosis [8,32].

hIAPP has been shown to drive pancreatic islet β-cell death through the secretion of proinflammatory cytokines by macrophages, particularly IL-1β, causing islet dysfunction and ultimately glucose intolerance [8,9,11,33]. Indeed, the blockade of IL-1 receptors (IL-1R) *in vivo* and *in vitro* is able to reduce the formation of amyloid deposits in hIAPP-Tg islets [7,9]. In keeping with these observations, our studies demonstrated that DICs cocultured with peritoneal macrophages and challenged with hIAPP exhibited increased β-cell apoptosis and a robust impairment of β-cell growth. Remarkably, these hIAPP-induced adverse effects on pancreatic β-cells were prevented when AAT was added to the cocultures. AAT has been shown to attenuate IL-1β secretion in monocytes [34] and to increase IL-1R antagonist (IL-1Ra) levels, the natural inhibitor of IL-1 [18,30,35]. In this context, IL-1Ra has been shown to inhibit the recruitment of macrophages in hIAPP expressing grafts [7]. AAT did not reduce the secretion of IL-1β by cultured peritoneal macrophages challenged with hIAPP. However, AAT increased the levels of secreted IL-1Ra in the medium of hIAPP-treated macrophages previously primed with LPS. Thus, AAT treatment may favor the secretion of this anti-inflammatory soluble factor, thereby contributing to a better resolution of inflammation. Indeed, the amyloid formation has been suggested to impair the balance between IL-1β and IL-1Ra in human islets [36], and AAT may benefit islet function in part by restoring a beneficial IL-1β/IL-1Ra ratio for β-cells.

AAT protected β-cells from secreted factors released by hIAPP-treated macrophages. This was in line with the observation that AAT also protects β-cells from exogenously administered proinflammatory cytokines. An effect of AAT on β-cell survival is supported by studies demonstrating that AAT is internalized and inhibits caspase-3 in MIN6 β-cells [21] and in lung alveolar cells [37]. Moreover, AAT has also been shown to potently suppress c-Jun N-terminal kinase (JNK) phosphorylation triggered by proinflammatory cytokines [19] in β-cells. Because the JNK pathway mediates β-cell apoptosis downstream of amyloid formation [[38], [39], [40]], we can hypothesize that AAT could also promote β-cell protection from hIAPP-induced cytotoxicity by suppressing this signaling pathway. It has also been suggested that AAT may exert its beneficial effects against cytokines through blockade of the action of TNFα-converting enzyme (TACE), thereby inhibiting the release of membrane TNFα in islets exposed to IL-1β and IFNγ [17]. However, our results demonstrate that AAT is able to improve β-cell survival in the presence of a cocktail of active proinflammatory cytokines including TNFα. Although more studies are required to elucidate the cellular mechanisms underlying the favorable actions of AAT against hIAPP-induced β-cell dysfunction, the fact that AAT restrains the cytotoxic actions of hIAPP and proinflammatory cytokines on pancreatic β-cells may have an impact on the inflammatory loop between β-cells and resident macrophages, thereby attenuating the progression of inflammation.

The IL-1β blockade has been shown to be effective in protecting hIAPP-Tg mice islets [9]. However, this cytokine also plays an important physiological role in regulating β-cell proliferation and insulin secretion [41,42]. In this context, macrophage-derived IL-1β participates in the postprandial stimulation of insulin secretion through the highly expressed IL-1 receptor on β-cells [41]. Moreover, IL-1β has been shown to potentiate insulin secretion and it has been suggested to signal islet compensation in response to metabolic demands [43]. Additionally, specific pancreatic deletion of IL-1R in db/db mice worsens glucose tolerance [44]. Thus, blocking IL-1β action may affect the capacity of β-cells to properly secrete insulin. In this regard, AAT treatment may provide an alternative strategy to attenuate the detrimental effects of proinflammatory molecules in pancreatic islets in T2D. Indeed, a potential AAT-based therapeutic strategy may provide additional advantages for T2D patients because serum AAT levels have been shown to be decreased in obese mice and human subjects [45]. This results in an imbalance between AAT and its target neutrophil elastase, which was shown to mediate insulin resistance in mice fed a high-fat diet [46]. Accordingly, the overexpression of AAT protected high-fat-diet-fed mice against body weight gain, insulin resistance, inflammation, and fatty liver [45]. Therefore, augmentation therapy with AAT in T2D subjects may have a double potential therapeutic impact by protecting pancreatic islets from hIAPP-induced islet dysfunction and by ameliorating obesity and insulin resistance. The fact that AAT has been proven to be a safe drug that has been used effectively for the treatment of pulmonary emphysema in patients with hereditary deficiency of AAT for decades [16] would facilitate the use of AAT for the treatment of T2D.

In summary, here, we demonstrate that treatment with the protease inhibitor AAT recovers glucose homeostasis in a mouse model overexpressing hIAPP in pancreatic β-cells. Furthermore, AAT exerts a protective effect on pancreatic β-cells against the cytotoxic action of hIAPP and proinflammatory cytokines. These results highlight the potential of AAT administration as a novel therapeutic approach to rescue pancreatic β-cell function for the treatment of T2D.

## Authors’ contributions

J. R.-C., J. M.-V, M. O., C. C., S. P., G. A.-V., D. D.-C., A. M., R. H., and M. C. contributed to the performance of experiments and data analysis. J. R.-C., A. N., and J.-M. S. wrote the manuscript. A. N. and J.-M. S. designed the study. All authors contributed to the discussion of results, reviewed the manuscript, and approved the final version of this manuscript.
