# Supplementary material for: Alpha1-antitrypsin ameliorates islet amyloid-induced glucose intolerance and β-cell dysfunction
Source: Mol Metab. 2020 Mar 27;37:100984. doi: 10.1016/j.molmet.2020.100984 (PMC7186564; doi:10.1016/j.molmet.2020.100984)
Supplement: Multimedia component 1 [file mmc1.pdf]

Supplementary Figure S1
Alpha1-antitrypsin ameliorates islet amyloid-induced glucose intolerance and β-cell dysfunction
Júlia Rodríguez-Comas et al.

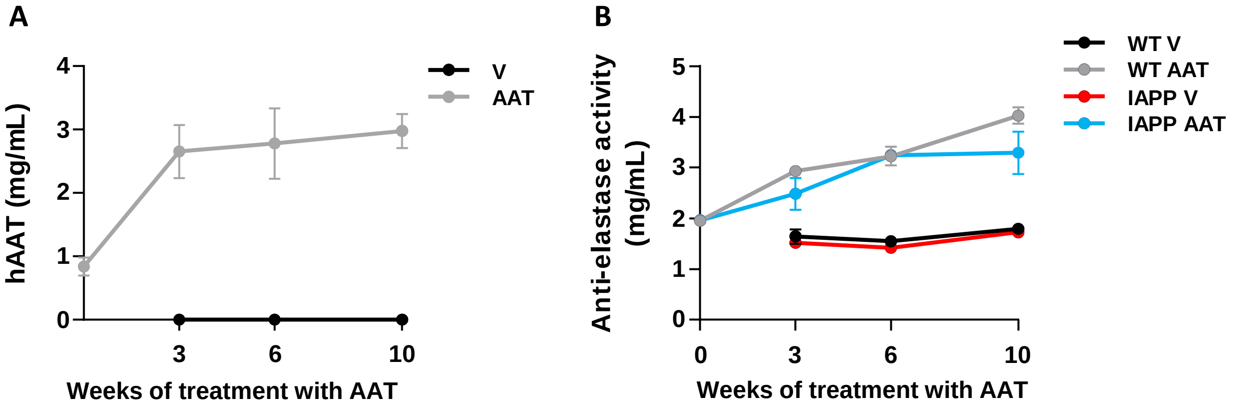

Supplementary Figure S1. (**A**) Plasma levels of human AAT (hAAT) in FVB/NJ mice injected intraperitoneally with human AAT (2-6 mg/mouse) or Vehicle (V) for 10 weeks. Plasma samples were collected 4 hours after the injection (n=7-12). (**B**) Anti-elastase activity in plasma samples of WT and hIAPP-Tg FVB/NJ mice treated intraperitoneally with AAT or Vehicle (V) from 6 to 16 weeks of age. The anti-elastase activity found in vehicle samples corresponds to endogenous murine anti-elastase activity. Increased levels of both hAAT and anti-elastase activity were observed in AAT-treated groups (AAT) compared to control groups (V) along the treatment course (n=3-8 per group).

Materials and Methods

Determination of human AAT levels: Human AAT levels in plasma samples collected from the tail were determined by sandwich ELISA. Briefly, at least two appropriate dilutions of plasma samples were incubated on 96-well plates previously coated with a hAAT antibody during 1 hour at 37ºC. After HRP-conjugated anti-human AAT and substrate addition, plates were read at 450 nm. Purified hAAT (Grifols) calibrated in front of an international standard (NIBSC) was used as standard.

Determination of anti-elastase activity: Anti-elastase activity in plasma samples collected from the tail was determined by enzymatic assay. Briefly, three appropriate dilutions of plasma samples were incubated with porcine pancreatic elastase on 96-well plates during 10 minutes at 25ºC. After substrate addition, plates were read at 405 nm. Purified hAAT (Grifols) calibrated in front of an international standard (NIBSC) was used as standard.
