# Supplementary material for: Alpha1-antitrypsin ameliorates islet amyloid-induced glucose intolerance and β-cell dysfunction
Source: Mol Metab. 2020 Mar 27;37:100984. doi: 10.1016/j.molmet.2020.100984 (PMC7186564; doi:10.1016/j.molmet.2020.100984)
Supplement: Multimedia component 2 [file mmc2.pdf]

Supplementary Figure S2
Alpha1-antitrypsin ameliorates islet amyloid-induced glucose intolerance and β-cell dysfunction
Júlia Rodríguez-Comas et al.

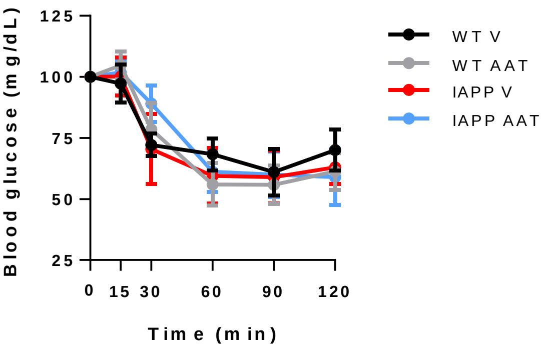

Supplementary Figure S2. Insulin tolerance test in AAT-treated and non-treated WT and hIAPP-Tg mice. 5h-fasted mice were injected intraperitoneally with human insulin solution (0.7 U/Kg). Glucose levels in tail vain blood samples were measured before insulin injection (baseline) and 15, 30, 60, 90 and 120 min after the injection. Values normalized to baseline glucose levels were analyzed and no significant differences were found between the four experimental groups (ANOVA). Results are presented as means ± SEM.
