# Supplementary material for: Alpha1-antitrypsin ameliorates islet amyloid-induced glucose intolerance and β-cell dysfunction
Source: Mol Metab. 2020 Mar 27;37:100984. doi: 10.1016/j.molmet.2020.100984 (PMC7186564; doi:10.1016/j.molmet.2020.100984)
Supplement: Multimedia component 3 [file mmc3.pdf]

Supplementary Figure S3
Alpha1-antitrypsin ameliorates islet amyloid-induced glucose intolerance and β-cell dysfunction
Júlia Rodríguez-Comas et al.

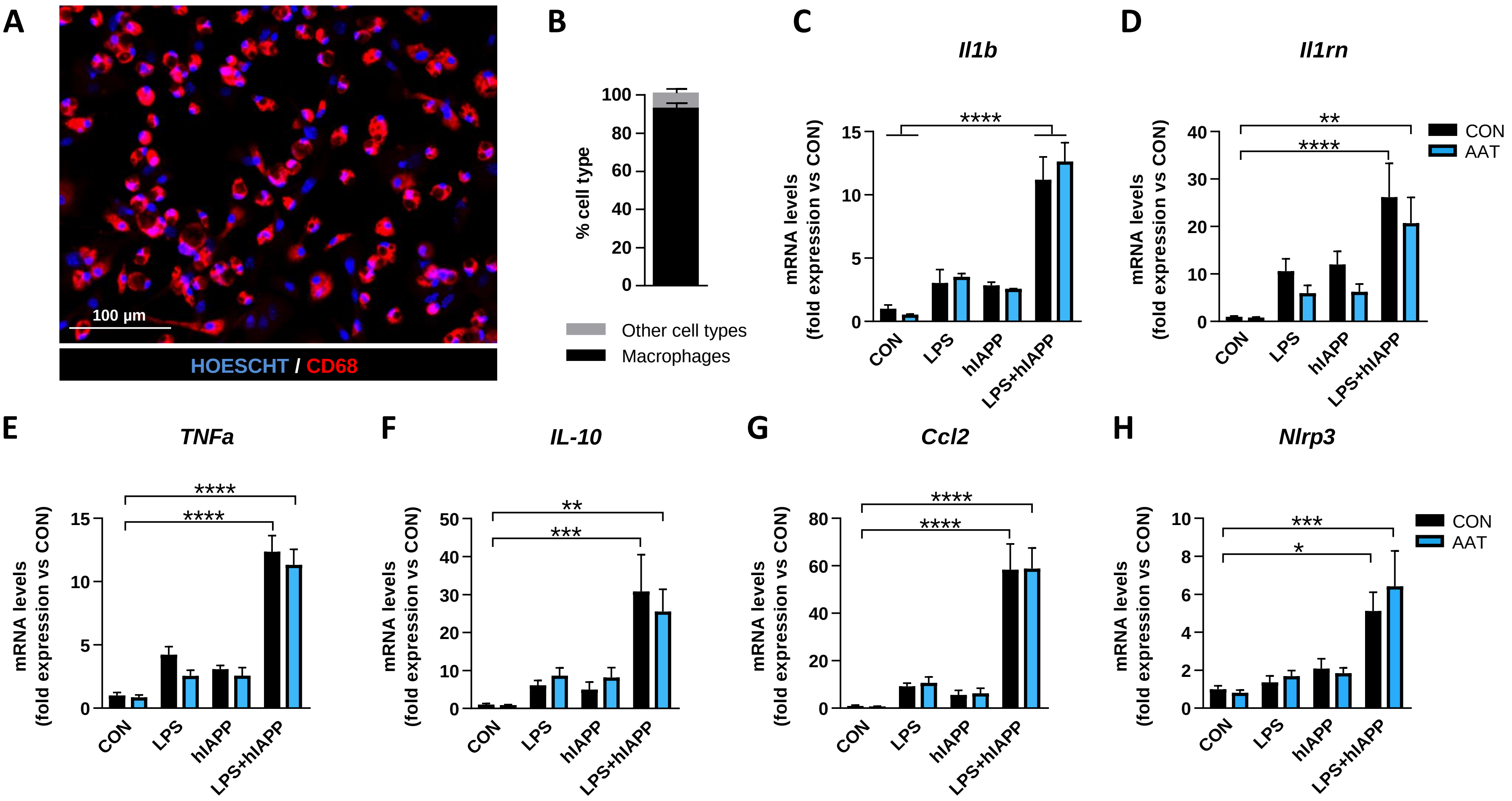

Supplementary Figure S3. (**A**) Immunostaining for CD68 (red) and Hoechst (blue) of isolated peritoneal macrophages. (**B**) Quantification of the percentage of macrophages over the total cell number present in the culture. (**C-H**) Gene expression analysis of (**C**) *Il1b*, (**D**) *Il1rn*, (**E**) *Tnfa*, (**F**) *Il10*, (**G**) *Ccl2*, and (**H**) *Nlrp3* of non-treated cultured peritoneal macrophages or treated with LPS (5 ng/mL), hIAPP (10 μM) or a combination of both, in the absence or presence of AAT. Gene expression data were normalized against *Hprt1* and are shown relative to non-treated macrophages (CON). Results are expressed as the mean ± SEM from three independent experiments. *p<0.05; **p<0.01; ***p<0.001; ****p<0.0001.
