# Supplementary material for: Alpha1-antitrypsin ameliorates islet amyloid-induced glucose intolerance and β-cell dysfunction
Source: Mol Metab. 2020 Mar 27;37:100984. doi: 10.1016/j.molmet.2020.100984 (PMC7186564; doi:10.1016/j.molmet.2020.100984)
Supplement: Multimedia component 4 [file mmc4.pdf]

Supplementary Table S1
Alpha1-antitrypsin ameliorates islet amyloid-induced glucose intolerance and β-cell dysfunction
Júlia Rodríguez-Comas et al.

Supplementary Table S1. Primer sequences used for gene expression analysis for qPCR.

Gene	Species	Fw	Rv
Mafa	Mouse	CAAGGAGGAGGTCATCCGAC	TCTCCAGAATGTGCCGCTG
Pdx1	Mouse	CCCCAGTTTACAAGCTCGCT	CTCGGTTCCATTCGGGAAAGG
Emr1	Mouse	GCCCTGAACATGCAACCTG	CTCCTCCACTAGATTCAAGTCCT
Il1b	Mouse	GCAACTGTTCCTGAACTCAACT	ATCTTTTGGGGTCCGTCAACT
Ddit3/Chop	Mouse	TCATCCCCAGGAAACGAAGAG	GCTTTGGGATGTGCGTGTG
Trib3	Mouse	CGTGGCACACTGCCACAAG	TCCAGGTTCTCCAGCACCAG
Fas	Mouse	TCTGGGCTGTCCTGCCTCT	ACGAACCCGCCTCCTCAG
Mpeg1	Mouse	GAGAGTGAAACAAAAGCCAGACA	GCTGCTCCAATTGCAAAACAG
Lyz1	Mouse	TGGAATGGATGGCTACCGTG	CCATAGTCGGTGCTTCGGTC
Atf3	Mouse	GTCCGGGCTCAGAATGGAC	CGTGCCACCTCTGCTTAGCT
Il1r	Mouse	TGTAAGTAATGCTGTCCTGGGCTG	ATGAGCCCCAGTAGCACTTTCA
Il1rn	Mouse	GTGTCCTGTTTAGCTCACCCAT	TATCCCAGATTCTGAAGGCTTGC
Tnfa	Mouse	CCCTCACACTCAGATCATCTTCT	GCTACGACGTGGGCTACAG
Il10	Mouse	GCGCTGTCATCGATTTCTCC	ATGGCCTTGTAGACACCTTGG
Ccl2	Mouse	TTAAAAACCTGGATCGGAACCAA	GCATTAGCTTCAGATTTACGGGT
Nlrp3	Mouse	ATTACCCGCCCGAGAAAGG	TCGCAGCAAAGATCCACACAG
Hprt1	Mouse	GGTTAAGCAGTACAGCCCCA	TCCAACACTTCGAGAGGTCC
